# Waves spontaneously generated by heterogeneity in oscillatory media

**DOI:** 10.1038/srep25177

**Published:** 2016-05-04

**Authors:** Xiaohua Cui, Xiaodong Huang, Gang Hu

**Affiliations:** 1 School of Systems Science, Beijing Normal University, Beijing 100875, P.R. China; 2 Department of Physics, South China University of Technology, Guangzhou 510641, P.R. China; 3 Department of physics, Beijing Normal University, Beijing 100075, P.R. China

## Abstract

Wave propagation is an important characteristic for pattern formation and pattern dynamics. To date, various waves in homogeneous media have been investigated extensively and have been understood to a great extent. However, the wave behaviors in heterogeneous media have been studied and understood much less. In this work, we investigate waves that are spontaneously generated in one-dimensional heterogeneous oscillatory media governed by complex Ginzburg-Landau equations; the heterogeneity is modeled by multiple interacting homogeneous media with different system control parameters. Rich behaviors can be observed by varying the control parameters of the systems, whereas the behavior is incomparably simple in the homogeneous cases. These diverse behaviors can be fully understood and physically explained well based on three aspects: dispersion relation curves, driving-response relations, and wave competition rules in homogeneous systems. Possible applications of heterogeneity-generated waves are anticipated.

Waves represent an important means of transferring energy in the natural world. In particular, waves in nonlinear extended systems have attracted a great deal of attention in recent decades due to the richness and complexity of their outputs, e.g., solitary waves in dissipative systems, excitable waves in cardiac tissues, calcium waves in cardiac myocytes, and phase waves in oscillatory systems. To reveal the basic properties, various properties of waves, such as the formation, propagation, competition and interaction of nonlinear waves in homogeneous media, have been discussed extensively and comprehensively^1^,^2^,^3^,^4^,^5^,^6^,^7^,^8^,^9^,^10^,^11^,^12^. Nevertheless, most of the materials used in contemporary life and industry are heterogeneous and multi-component. Heterogeneity can thus describe realistic media much better; however, such media have been investigated and are understood much less due to their extremely complex dynamics and greater richness of the outputs^13^,^14^,^15^,^16^,^17^.

The complex Ginzburg-Landau equation (CGLE) is a model widely used for investigating nonlinear wave properties of oscillatory media because it provides a universal description of extended systems in the vicinity of a Hopf bifurcation from a homogeneous stationary state^4^,^18^,^19^. In a spatially extended CGLE system, we can observe great diversities of wave propagation and competition patterns. Rich behaviors of homogeneous CGLEs have been well understood^6^,^19^,^20^,^21^. First, in a homogeneous CGLE, if we pace the system on a boundary, we can observe normal waves (NWs, waves propagating away from sources) and anti-waves (AWs, waves propagating toward sources)^21^,^22^, depending on the pacing frequencies and parameter sets. Second, if we pace the system on both boundaries with different frequencies, two trains of waves with different frequencies, which compete in the homogeneous medium, can be generated. The rules for the competition results can be stated briefly as follows^17^,^18^,^22^,^23^,^24^,^25^:


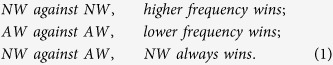


Moreover, without external pacing, the autonomous homogeneous CGLE with the no-flux boundary condition can support spiral and turbulent waves (*N*D media, *N* ≥ 2) and homogeneous oscillation (*N*D media, *N* ≥ 1), according to the initial variable conditions and parameter sets. For the simplest 1D CGLE with no flux boundary condition, the only pattern persistently surviving is simply homogeneous oscillation. Here, we focus on the analysis on the effects of heterogeneity which turns to be very complicated.

Some novel results have been observed in two-medium CGLE systems, such as interface-selected waves (ISW)^26^ and circle interface-selected waves^12^. ISWs are wave trains automatically generated from the interface of two different types of media, if one medium supports NWs while the other supports AWs. This phenomenon was first reported in a 1D inhomogeneous system modeled by CGLE in^26^. This type of waves can take part in the competition and may dominate the whole inhomogeneous system in some cases.

In this paper, we focus on the spontaneous generation of waves in heterogeneous CGLE media. As a simplest model, we consider 1D CGLE systems consisting of multiple different homogeneous submedia with interfaces between neighbor submedia. Rich patterns and greatly diverse phenomena have been observed for this rather simple heterogeneity, as follows: 1) multiple natural oscillations coexist in the multi-submedium with their own natural frequencies; 2) all submedia oscillate with the natural frequency of a single submedium, which dominates all CGLE multiple subsystems; 3) some submedia oscillate with their own natural frequency, whereas others show traveling waves; and 4) identical traveling waves run throughout the whole multi-media and the interfaces seem to be transparent. Some of these observations are totally beyond intuitive expectation. The mechanisms underlying these features can be fully understood and well predicted, according to three physical principal relations: (1) the dispersion relations of submedia, (2) the driving-response curves of homogeneous media under external pacing, and (3) the competition laws Eq. (1) in homogeneous media. Using these simple rules, the very rich wave behaviors and complex spatio-temporal patterns of the inhomogeneous CGLE systems can be clearly classified and well predicted. The understanding and explanation of such interesting phenomena for simple heterogeneous systems are expected to be extendable to more complicated realistic heterogeneous oscillatory systems and to help us understand heterogeneity-induced complexity, which is of great significance in practice.

## Results

### Model Description

We consider a one-dimensional (1D) oscillatory system modeled by using CGLE, i.e.,


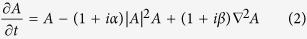


with the complex variable *A*(*x*, *t*) being the order parameter at a Hopf bifurcation^4^,^18^,^19^. It is well known that a homogeneous CGLE system has an inherent oscillation with natural frequency *ω*_0_ = *α*, and in appreciable regions of the (*α*, *β*) parameter space, NWs or AWs can be generated by applying external pacing with proper frequencies^19^,^22^. We study a 1D CGLE system of length *NL* (*N* ≥ 2). The system is divided into *N* submedia with different sets of parameters, and each submedium is homogeneous, with length *L*. These submedia are denoted as *M*_*i*_ (*i* = 1, …, *N*). Each two neighbor submedia have an interface point in between. Eq. (1) is now replaced by


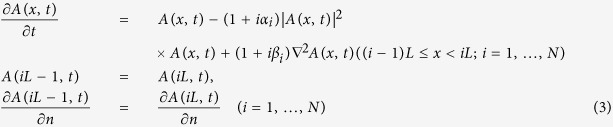


It is emphasized that Eq. (2) is a generic form of amplitude equation of any extended oscillatory media around a Hopf bifurcation from homogeneous steady state. A general derivation to multi-medium CGLE has been presented in ref. 26 where the generic CGLE contains more control parameters than Eq. (3). Here, we consider the simplified form of Eq. (3) without losing any essential features of heterogeneity-induced diversity of pattern formations.

### Wave Patterns Observed in Two-medium CGLEs

**Figure 1:**
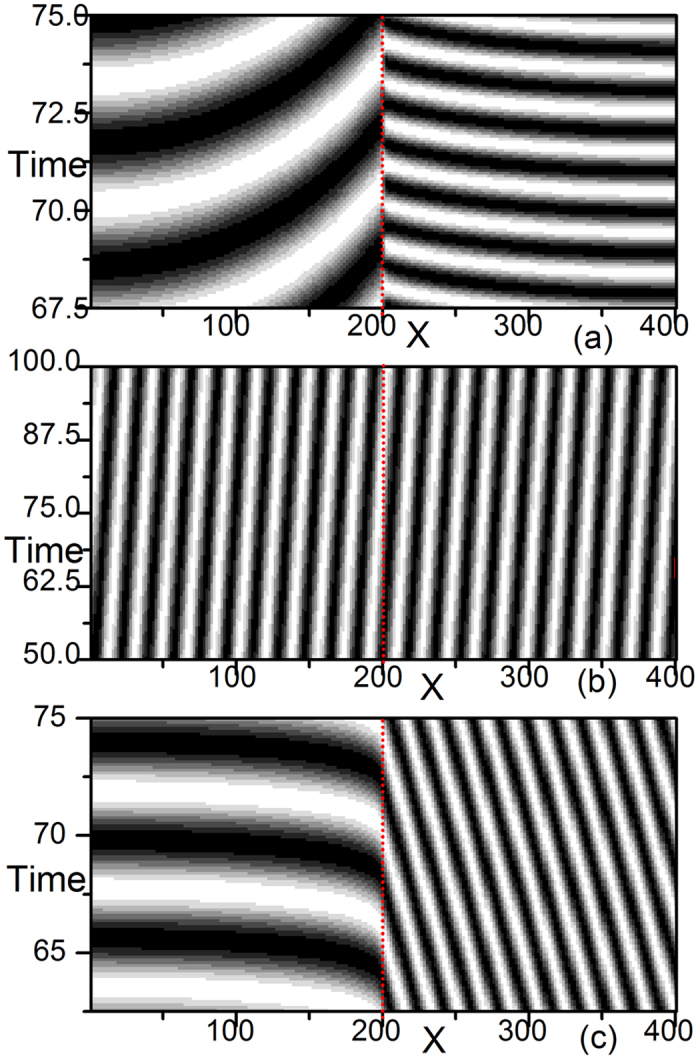
Spatio-temporal patterns of the real part of *A*(*x*, *t*), ReA(x, t), for a 1D two-submedium CGLE system (Eq. (3)). In this and all following figures, the no-flux (Neumann) boundary condition is applied and the time step Δ*t* = 0.005 and space step Δ*x* = 1.0 are used. A 2 × 200 chain with an interface (at x = 200, marked by red dots) in the middle is used for numerical simulations. The left and right submedia are CGLE systems with parameters *α*_1_, *β*_1_ (0 ≤ *x* ≤ *L*) and *α*_2_, *β*_2_ (*L* < *x* ≤ 2*L*), respectively. (**a**) *α*_1_ = 0.2, *β*_1_ = 0.3; *α*_2_ = −0.6, *β*_2_ = −1.0. Two natural oscillations with natural frequencies (*ω*_1_ = *α*_1_, *ω*_2_ = *α*_2_) coexist. Each natural oscillation dominates its own submedium, and none can spread over the interface. The interface is clearly visible in the contour pattern. (**b**) *α*_1_ = −0.15, *β*_1_ = 0.6; *α*_2_ = 0.2, *β*_2_ = −1.5. None of the natural oscillations wins the competition. A plane wave train with a new frequency *ω*_*int*_ = 0.04 ≠ *α*_1_, *α*_2_ dominates the entire 2*L* system. No interface can be observed. (**c**) *α*_1_ = −0.15, *β*_1_ = 1.0; *α*_2_ = −0.3, *β*_2_ = 1.0. The homogeneous oscillation with natural frequency *ω* = *α*_1_ dominates the *M*_1_ submedium, and it crosses over the interface, invades and dominates the other submedium *M*_2_. The two-medium system has the same frequency *ω*_1_ = *ω*_2_ = *α*_1_ in both submedia, and we observe a homogeneous oscillation in one submedium (*M*_1_), and a plane wave train in the other (*M*_2_).

We are now interested in how the heterogeneity can significantly influence the inherent dynamics, but no external pacing is involved. Though our model introduces heterogeneity in a simple way, this simple inhomogeneity produces pattern formation behaviors incomparably more complex than those of homogeneous systems, as we see in this section (*N* = 2, systems composed of two homogenous submedia, and *N* ≥ 3, systems composed of multiple homogenous submedia). It is well known that the output of homogeneous 1D CGLE is nothing but a homogeneous oscillation with natural frequency *ω*_0_ = *α*. We study the two-medium CGLE Eq. (3) (*N* = 2) with different parameter set arrangements. Random initial conditions are adopted for all numerical computations, and all characteristically different features in the asymptotic states are given in Fig. 1.

In Fig. 1(a), we observe the coexistence of two oscillations with different frequencies. Each oscillation, which has the natural frequency of the corresponding submedium, dominates its own domain, *ω*_0_(*M*_1_) = *α*_1_, *ω*_0_(*M*_2_) = *α*_2_, and none can spread over the interface. The interface is clearly visible in contour patterns. In Fig. 1(b), none of the natural oscillations wins the competition; a homogeneous plane wave train with a new frequency *ω*_*int*_ ≠ *α*_1_, *α*_2_ dominates the entire 2*L* system. The wave train is homogeneous in the whole inhomogeneous system, and the interface seems to be transparent and not visible. In Fig. 1(c), one natural oscillation exists in its own submedium, and its oscillation spreads over the interface and dominates the other submedium. The two-medium system has the same frequency in both submedia; we observe homogeneous oscillation with a natural frequency *α*_*i*_ in one submedium and a wave train driven by the same natural frequency *α*_*i*_ in the other. In Fig. S1 of the Supplemental Material, we plot a diagram showing the regimes where patterns in Fig. 1(a–c) can be observed. It is clear that all these patterns appear robustly in a wide parameter area.

### Wave Patterns Observed in Multi-medium CGLEs

**Figure 2:**
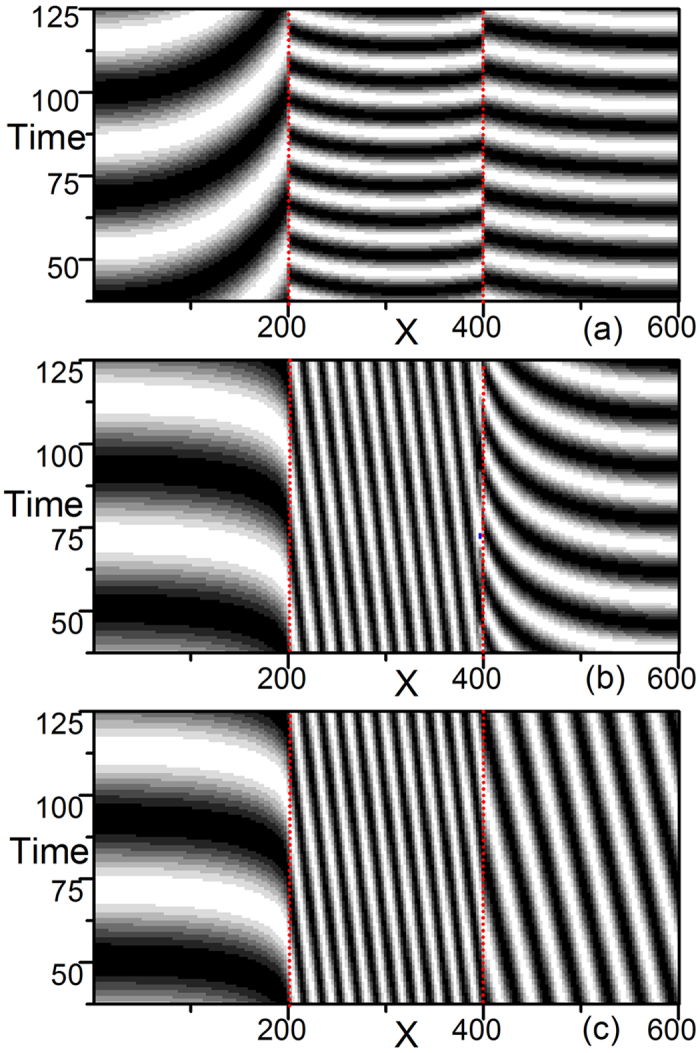
Spatio-temporal patterns of the real part of *A*(*x*, *t*) for a 1D three-submedium CGLE system (Eq. (3)). A 3 × 200 chain with two interfaces (at x = 200 and 400, marked by red dots) is used for numerical simulations. (**a**) *α*_1_ = 0.2, *β*_1_ = 0.3; *α*_2_ = −0.6, *β*_2_ = −1.0; and *α*_3_ = −0.5, *β*_3_ = −0.2. Three natural oscillations with natural frequencies coexist. Each natural oscillation dominates its own submedium, and none can spread over the interface. The interfaces are visible in the contour pattern. (**b**) *α*_1_ = −0.15, *β*_1_ = 1.0; *α*_2_ = −0.3, *β*_2_ = 1.0; and *α*_3_ = −0.4, *β*_3_ = −0.6. A coexistence of two natural oscillations (in *M*_1_ and *M*_3_) and a wave train (in *M*_2_) is observed. The natural oscillation in *M*_1_ dominates its own submedium, spreads over the interface to *M*_2_, and conquers *M*_2_. Meanwhile, the natural oscillation in *M*_3_ maintains its own submedium. (**c**) *α*_1_ = −0.15, *β*_1_ = 1.0; *α*_2_ = −0.3, *β*_2_ = 1.0; and *α*_3_ = −0.2, *β*_3_ = 1.1. The natural oscillation in *M*_1_ conquers *M*_2_ and *M*_3_; we observe a natural oscillation in *M*_1_ and wave trains in *M*_2_ and *M*_3_, and all three submedia oscillate with the same frequency *ω*_1_ = *ω*_2_ = *ω*_3_ = *α*_1_ and with different wave numbers. Both interfaces are visible.

When inhomogeneous systems are composed of multiple homogeneous submedia (*N* ≥ 3), there are multiple natural oscillations, and possibly more than one ISW train is involved in wave competitions. The resulting pattern formation is much more complicated. Taking systems of *N* = 3 as examples, in Fig. 2, we observe the coexistence of distinctive homogeneous oscillations in three submedia (Fig. 2(a), the frequencies of three domains are obviously different and are equal to their own natural frequencies *ω*_*i*_ = *α*_*i*_, *i* = 1, 2, 3), the coexistence of both distinctive homogeneous oscillations in two submedia and a traveling wave train in the other (Fig. 2(b), homogeneous oscillations in the left and right domains and a running wave train from right to left in the middle submedium; the frequencies in the left two domains are the same and are different from the frequency in the right domain), and the coexistence of natural oscillation and traveling wave trains (Fig. 2(c), homogeneous oscillation in the left domain and running wave trains in the other domains; the frequencies of the three domains are the same).

**Figure 3:**
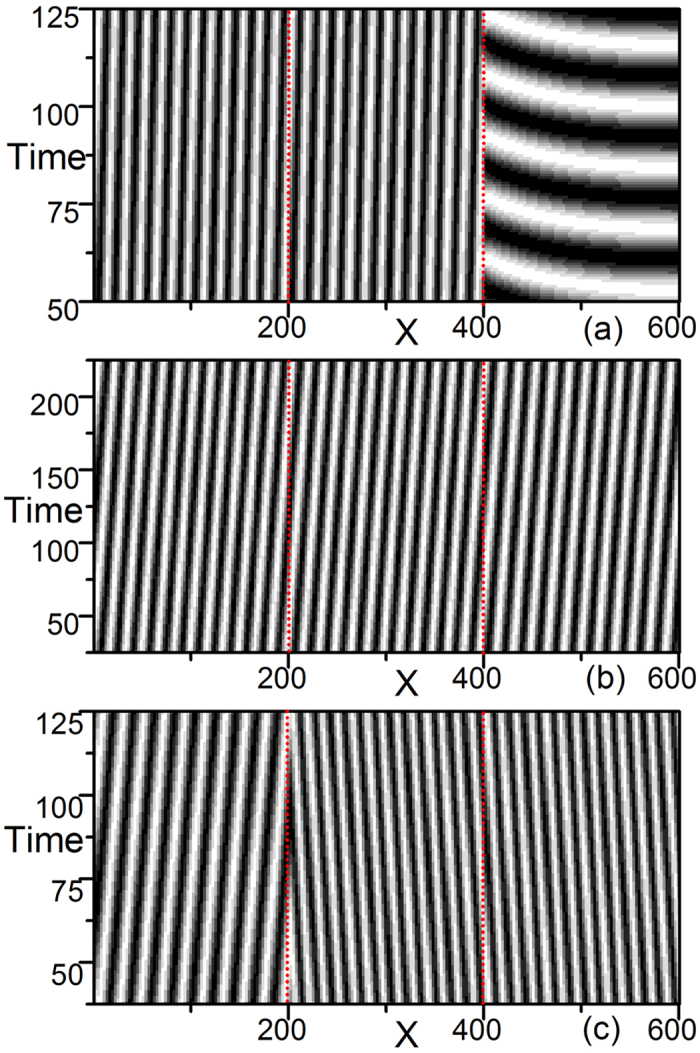
The same as Fig. 2 but with different parameter sets. (**a**) *α*_1_ = −0.15, *β* = 0.6; *α*_2_ = 0.2, *β*_2_ = −1.5; and *α*_3_ = 0.4, *β*_3_ = 0.6. A coexistence of the natural oscillation and a wave train is observed. A plane wave train with a new frequency *ω*_*int*_ = 0.04 ≠ *α*_1_, *α*_2_ dominates the 2*L* system (*M*_1_ and *M*_2_), and the natural oscillation with *α*_3_ dominates its own submedia. (**b**) *α*_1_ = −0.15, *β*_1_ = 0.6; *α*_2_ = 0.2, *β*_2_ = −1.5; and *α*_3_ = 0.1, *β*_3_ = −0.4. A plane wave train dominates all three submedia. The wave numbers in *M*_1_ and *M*_2_ are equal, and both are different from *M*_3_. (**c**) *α*_1_ = −0.15, *β*_1_ = 0.6; *α*_2_ = 0.2, *β*_2_ = −1.5; and *α*_3_ = −0.3, *β*_3_ = 1.0. We observe two wave trains in the system; one wave train in *M*_1_ and another in *M*_2_ and *M*_3_. The interface is visible between *M*_1_ and *M*_2_ and transparent between *M*_2_ and *M*_3_. All three submedia oscillate with the same frequency *ω* = 0.08 ≠ *α*_1_, *α*_2_, *α*_3_.

In Fig. 3, we observe the coexistence of ISWs and natural oscillation (Fig. 3(a), with ISW trains dominating the left two domains and homogeneous natural oscillation dominating the right domain), wave trains in three domains (Fig. 3(b), plane wave trains dominate the whole three-medium system, and the wave numbers are different in the three submedia; *k*^2^(*M*_1_) = *k*^2^(*M*_2_) ≠ *k*^2^(*M*_3_)), and also wave trains in the three domains (Fig. 3(c), the frequencies of the three submedia are the same, but the directions of wave propagation are obviously different from those in Fig. 3(b)).

Because a 1D homogeneous CGLE medium with a no-flux boundary can produce only homogeneous oscillations with natural frequency *ω*_0_ = *α* robustly for arbitrary random initial conditions and arbitrary parameter arrangements, the diverse patterns of Figs 1, 2, 3 produced by the simplest heterogeneity of interfaces of different homogeneous submedia are interesting and significant. A number of problems are raised: what physical mechanisms are responsible for producing the different patterns of Figs 1, 2, 3 and how different parameter distributions are related to these distinctive pattern formations. We answer these problems in the following section.

### Mechanisms Underlying the Diversity of Pattern Formations in Two-medium Systems

The phenomena of Fig. 1 look complicated and contradictory. However, as we will see, all of the complexity can be understood and well explained by jointly considering three items: (i) the dispersion relations of CGLE media, (ii) the driving-response curves under external pacings, and (iii) the competition rules of Eq. (1) in any homogeneous media. For the wave competitions (iii), different waves can be involved depending on different physical situations, such as two natural oscillations, interface-selected waves (ISWs^26^, which we will see below), and other driving-generated waves.

First, we briefly introduce the existence of ISWs. If two dispersion relation curves of two neighbor submedia have slopes with opposite signs in the *ω* − *k*^2^ plane and intersect each other, an ISW can definitely exist. Whenever this ISW appears, it can compete with the natural oscillations in both submedia and win the competitions against the two natural oscillations on both sides according to Eq. (1). ISW can exist, dominate both submedia, and generate a wave train with the same frequency that propagates in the whole two-medium system.

**Figure 4:**
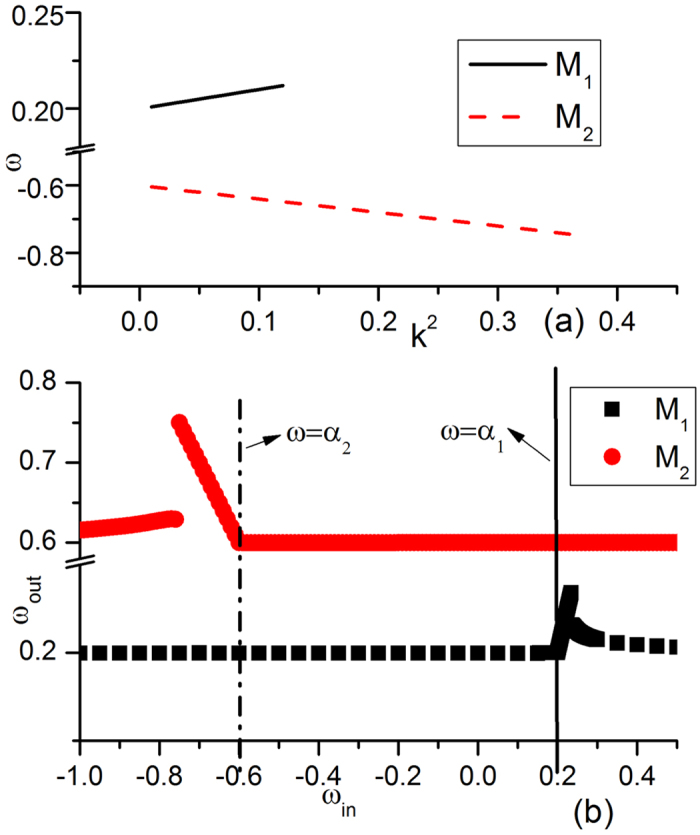
Dispersion relation curves and driving-response curves of Fig. 1(a) plotted for the two submedia *M*_1_, *M*_2_. All parameters are taken from Fig. 1(a). (**a**) Dispersion relation curves of the two submedia. One (*M*_1_) has a positive slope, the other (*M*_2_) has a negative slope, and the two curves have no intersection. (**b**) Driving-response curves of *M*_1_ and *M*_2_ under different driving frequencies. The x-axis is the frequency of external driving and the y-axis is the frequency of the output wave train. Here, the natural frequency (*α*_1_ for *M*_1_, *α*_2_ for *M*_2_) is not allowed by the other submedium (*α*_1_ not allowed by *M*_2_ and *α*_2_ not allowedy by *M*_1_) in the driving-response curves. Therefore, both submedia (*M*_1_, *M*_2_) are not driven by the other natural frequencies (*α*_2_, *α*_1_) and can only keep their own homogeneous oscillations with their own natural frequencies (*α*_1_, *α*_2_) in their own submedia, as shown in Fig. 1(a).

Now let us try to understand the mechanisms underlying the complexity of Fig. 1 by considering in detail the competitions of wave trains from different wave sources. In Fig. 4, we plot the dispersion relation curves and driving-response curves, with all parameters being taken from Fig. 1(a). The dispersion relation curves of two submedia (Fig. 4(a)) have slopes with opposite signs in the *ω* − *k*^2^ plane. One (*M*_1_) has a positive slope, and the other (*M*_2_) has a negative slope, but the two curves have no intersection. Thus, no ISW exists. Figure 4(b) shows the driving-response curves of *M*_1_ and *M*_2_ under different driving frequencies. From the figure, it is clear that the natural frequency *α*_1_ of *M*_1_ (*α*_2_ of *M*_2_) is not allowed by the other submedium *M*_2_ (*M*_1_) in the driving-response curves. Therefore, both submedia (*M*_1_, *M*_2_) are not driven by the other natural frequencies (*α*_2_, *α*_1_), and they can only keep their own homogeneous oscillations with their own natural frequencies (*α*_1_, *α*_2_), as shown in Fig. 1(a).

**Figure 5:**
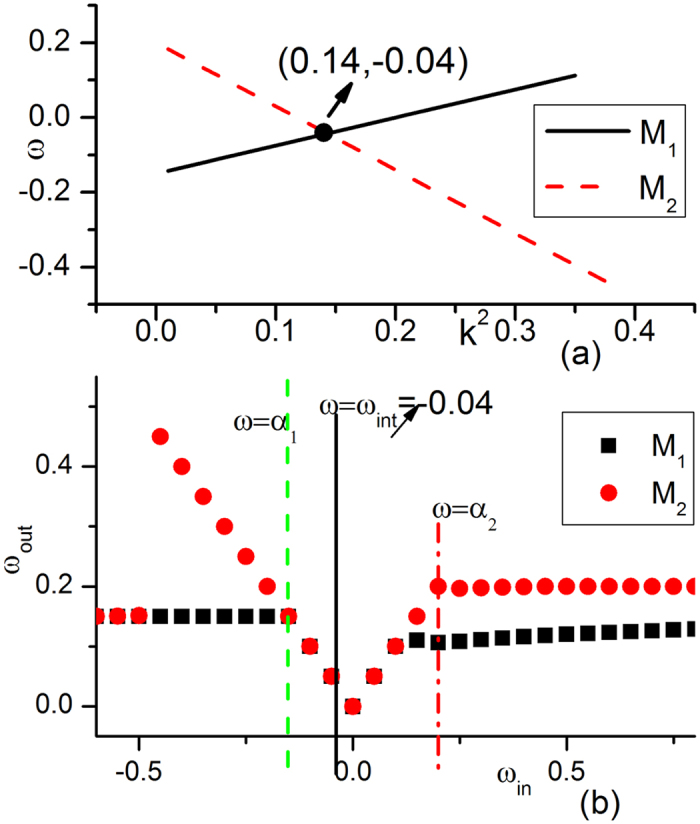
Dispersion relation curves and driving-response curves of system Fig. 1(b). (**a**) Dispersion relation curves of *M*_1_ and *M*_2_ with one (*M*_1_) having a positive slope and the other (*M*_2_), a negative slope. (**b**) Driving-response curves of *M*_1_ and *M*_2_. In (**a**), there is an intersection in the dispersion relation curves at *ω*_*int*_ = −0.04, 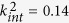. The interface can generate a plane wave train with frequency *ω* = *ω*_*int*_ and wave number 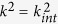 called interface-selected waves (ISW,^26^). Moreover, ISW is AW in *M*_1_ and NW in *M*_2_ according to Eq. (5). Due to the competition rules of Eq. (1), ISW can defeat the oscillation of natural frequency *α*_1_ in *M*_1_ and that with frequency *α*_2_ in *M*_2_, finally dominate the whole two-medium system.

In Fig. 5, the dispersion relation curves and driving-response curves of the system with the parameters of Fig. 1(b) are plotted. The two dispersion relation curves of *M*_1_ and *M*_2_ have slopes with opposite signs (Fig. 5(a), *M*_1_ has a positive slope, and *M*_2_ has a negative slope). Now, the two curves have an intersection at *ω*_*int*_ = −0.04, 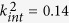. In the driving-response curves (Fig. 5(b)), we find that this driving-frequency is allowed by both *M*_1_ and *M*_2_. An ISW now exists, i.e., the interface can generate a plane wave train with frequency *ω* = *ω*_*int*_ and wave number 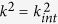 called the interface-selected waves (ISWs,^26^). Moreover, the ISW is AW in *M*_1_ and NW in *M*_2_ according to Eq. (5). Due to the competition rules of Eq. (1), ISW can defeat the oscillation of natural frequency *α*_1_ in *M*_1_ (*ω*_*int*_ < *α*_1_; lower frequency wins for two AWs) as well as that with frequency *α*_2_ in *M*_2_ (the natural oscillation is AW, whereas ISW is NW; NW defeats AW); finally, it dominates the whole two-medium system. Therefore, one can observe a uniform plane wave train propagating throughout the two-medium system in Fig. 1(b).

**Figure 6:**
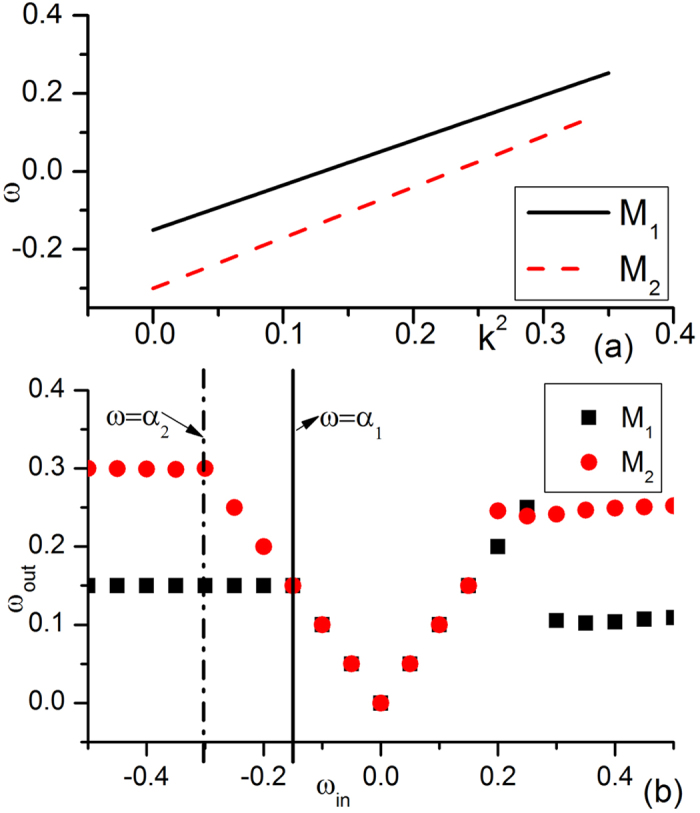
Dispersion relation curves and driving-response curves of Fig. 1(c). (**a**) Dispersion relation curves of *M*_1_ and *M*_2_. The slopes of the two curves have the same sign (both slopes are now positive). Here, no ISW exists and the competition between the two natural oscillations is crucial (unlike Fig. 1(a,b)). (**b**) Driving-response curves for *M*_1_ and *M*_2_. In (**a**), *α*_1_ is allowed by *M*_2_, and external pacing with *α*_1_ will generate a wave train with the same frequency in *M*_2_ and defeat the natural oscillation of *α*_2_ in both submedia *M*_1_ and *M*_2_. Finally, the oscillation of frequency *α*_1_ dominates its own submedium and conquers the other submedium with the same frequency, as shown in Fig. 1(c).

In Fig. 6, the dispersion relation curves and driving-response curves with the parameters of Fig. 1(c) are plotted. The slopes of the two dispersion relation curves have the same sign (both slopes are positive; see Fig. 6(a)). Now, unlike Fig. 1(b), no ISW exists or joins the wave competition. The natural oscillations of *M*_1_ and *M*_2_ are the only competition participants. Figure 6(b) shows the driving-response curves for *M*_1_ and *M*_2_. *α*_1_ is allowed by *M*_2_, and external pacing with *α*_1_ will generate a wave train with the same frequency *ω* = *α*_1_ in *M*_2_ and defeat natural oscillation *α*_2_ according to the rule of Eq. (1) (both are AWs, |*α*_1_| < |*α*_2_|), while *α*_2_ cannot generate a wave train in *M*_1_. Thus, natural oscillation *α*_1_ dominates its own submedium and conquers the other submedium with the same frequency, as shown in Fig. 1(c).

From the analysis in Figs 4, 5, 6, we can identify three typical types of patterns in Fig. 1 in two-submedium CGLE systems due to interface competition.

Competitions at Interface

Type A: When the two dispersion relation curves of the two submedia do not overlap in their frequency ranges, namely, a frequency allowed by one submedium is not allowed by the other submedium, the motions in two submedia do not invade each other and we can observe coexisting homogeneous oscillations in the two domains with their own natural frequencies *ω*_1_ = *α*_1_, *ω*_2_ = *α*_2_, as shown in Fig. 1(a).

Type B: If the two dispersion relation curves of the two submedia intersect with each other at a point (*ω*_*int*_, 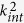) and they have slopes with opposite signs, an ISW with *ω* = *ω*_*int*_, 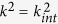 appears. ISW dominates both submedia, as shown in Fig. 1(b).

Type C: When the two dispersion curves have slopes with the same signs and overlap in frequency regions, the two homogeneous oscillations compete. The one who can win the competitions in the two submedia dominates not only its own submedia but also in the other submedium by crosing the interface. Then, one observes homogeneous oscillation in one submedium (*M*_*i*_) with the natural frequency *ω*_*i*_ = *α*_*i*_ and an inhomogeneous plane wave train in the other submedium *M*_*j*≠*i*_ with the same frequency *α*_*i*_, as shown in Fig. 1(c).

### Mechanisms Underlying the Diversity of Pattern Formations in Multi-medium Systems

The phenomena observed in systems composed of three submedia are much more complex and diverse than that in the two-medium system. The mechanism underlying the complexity is, however, still simple and understandable. All the analyses are based on the competitions between various natural oscillations and ISWs, concretely, between two natural oscillations of neighbor submedia and related ISW and between two neighbor ISWs. The conclusion summarized for the two-medium system can be applied directly with slight extensions.

For the parameter set of Fig. 2(a), the three dispersion relation curves of the submedia do not overlap in the frequency region. It is easy to judge that both of the competitions around the two interfaces are Type A, none of the natural oscillations can be allowed by the guest submedia, and three natural oscillations coexist.

**Figure 7:**
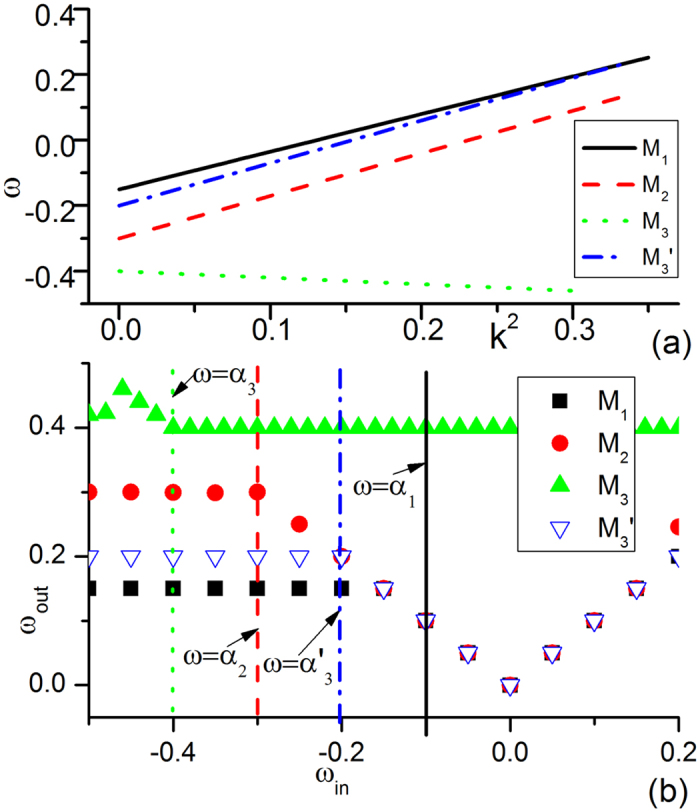
Dispersion relation curves and driving-response curves of systems in Fig. 2(b,c). Figure 2(b) has *M*_1_: *α*_1_ = −0.15, *β*_1_ = 1.0; *M*_2_: *α*_2_ = −0.3, *β*_2_ = 1.0; *M*_3_: *α*_3_ = −0.4, *β*_3_ = −0.6. Figure 2(c) has the same *M*_1_ and *M*_2_ as (**b**), and *M*_3_: 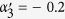, 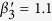. (**a**) Dispersion relation curves of submedia (*M*_1_, *M*_2_, *M*_3_, 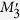). These submedia (*M*_1_, *M*_2_, 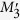) have slopes with the same positive signs and *M*_1_, *M*_2_, 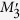 overlap in the frequency regions. (**b**) Driving-response curves for all these submedia. *α*_1_ is allowed by *M*_2_ and 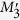 but not by *M*_3_. The natural oscillation *α*_1_ can dominate *M*_2_ (Type C) and can conquer 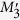 (Type C), producing the pattern in Fig. 2(c), but it cannot invade *M*_3_ (*α*_1_ is not allowed by *M*_3_), yielding the pattern in Fig. 2(b) (Type A).

In Fig. 7, the dispersion relation curves and driving-response curves of the systems in Fig. 2(b,c) are plotted. The systems of Fig. 2(b,c) have the same submedia *M*_1_ and *M*_2_ and different *M*_3_. In Fig. 7, the third submedia of Fig. 2(b,c) are denoted by *M*_3_ and 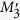, respectively, for distinction. Now, *M*_1_, *M*_2_ and 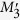 have slopes with the same positive signs, and overlap in the frequency regions. *α*_1_ and the oscillation of frequency *α*_1_ can defeat those with frequency *α*_2_ in *M*_2_ and *α*_3_ in 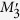. Therefore, in Fig. 5(c), the natural oscillation of the frequency of *ω* = *α*_1_ will dominate *M*_2_ (Type C) and will conquer 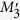 (Type C). However, in Fig. 5(b), the oscillation of *α*_1_ cannot spread to *M*_3_ (Type A) and produce the pattern of coexisting frequencies of *ω* = *α*_1_ in *M*_1_, *M*_2_ and *ω* = *α*_3_ in *M*_3_.

The above ideas can be fully applied to the patterns of Fig. 3. In the Supplemental Material (Figs S2 and S3), we explain the diverse behaviors of these results with similar spirits, case by case, according to all of the patterns shown in Fig. 3.

## Discussion

The above explanations are made case by case and look rather complicated, with too much detail. It would be desirable to summarize the mechanisms using some simple and convincing principles that universally govern the diverse results of the competitions. These compact principles do exist, and they are based on the three above-mentioned aspects, i.e., (i) the dispersion relations; (ii) the driving-response curves under external pacings; and (iii) the competition rules of Eq. (1), as well as three different types of wave trains, i.e., (1) the natural oscillations; (2) ISWs; (3) the driving-response of wave trains generated by natural oscillations or ISWs.

By considering the above three items and the three different types of waves involved in competitions and by considering the combinations of the above A, B, C pattern formations, the process of pattern formation in multi-medium CGLE systems can be summarized as follows:

1. Treat the multi-submedium system as two-medium systems, and consider Competitions at Interfaces ⊕ Competitions in Submedia
  - study all interfaces between submedium *M*_*i*_ and *M*_*i*+1_ (*i* = 1, 2, …, *N* − 1) and identify their types (A or B or C).
  - Investigate the wave competitions on the *i*_*th*_, (*i* = 1, 2, …, *N*) submedium with known types of a) and determine the winner of wave competitions according only to the rules of Eq. (1).
2. Repeat a) and b) of step 1 with the winners considered in step 1.
3. Repeat step 2 until a pattern of the whole *N* submedia becomes stable.

The steps and relevant items here are rather simple, but the results of Figs 1(a–c)–3(a–c) look rather complicated. The complexity of the results is due to the rich and diverse combinations of the conditions in the items, but the complexity can also be comprehensively classified based on these simple items.

**Figure 8:**
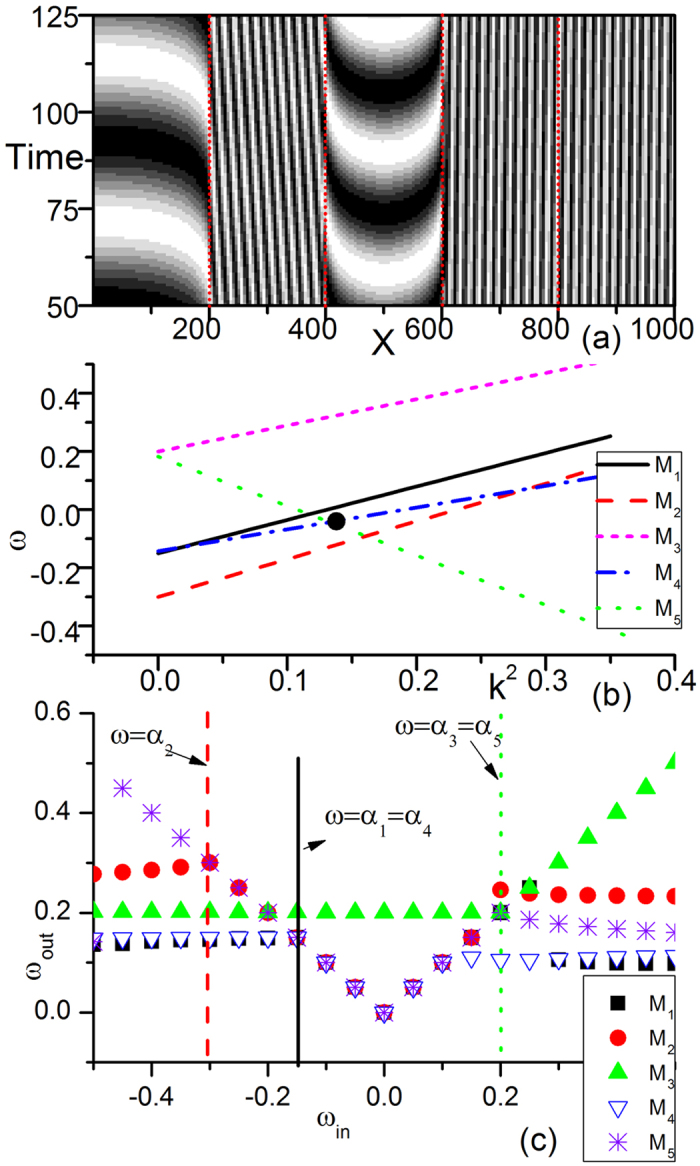
A 5 × 200 chain with four interfaces at *x* = 200, 400, 600, 800 (marked by red dots) is used for numerical simulations. All the submedia are CGLE systems with parameters *α*_*i*_, *β*_*i*_, (*i* = 1, 2, …, 5), respectively. *α*_1_ = −0.15, *β*_1_ = 1.0; *α*_2_ = −0.3, *β*_2_ = 1.0; *α*_3_ = 0.2, *β*_3_ = 1.1; *α*_4_ = −0.15, *β*_4_ = 0.6; and *α*_5_ = 0.2, *β*_5_ = −1.5. (**a**) Spatio-temporal patterns of the real part of *A*(*x*, *y*) for the 1D five-submedium CGLE system (Eq. (3)). We observe the coexistence of natural oscillations in *M*_1_ and *M*_3_ and wave trains in the other three submedia. The interface between *M*_4_ and *M*_5_ is transparent, while all others are visible. (**b**) Dispersion relation curves of these submedia. (**c**) Driving-response curves for the five submedia. The competition around the interface between (*M*_1_, *M*_2_) is of Type C (*α*_1_ dominates its own submedium and invades *M*_2_); around interfaces (*M*_2_, *M*_3_) and (*M*_3_, *M*_4_), Type A (coexistence of two natural oscillations); around interface (*M*_4_ and *M*_5_), Type B (ISW dominates both *M*_4_ and *M*_5_). Now, in some submedia (*M*_2_ and *M*_4_), there are two winners; the two winners should compete further according to the rules of Eq. (1). Natural oscillation *α*_1_ is left in *M*_2_ and ISW survives in *M*_4_. The competition around some interfaces (interfaces (*M*_2_, *M*_3_), (*M*_3_, *M*_4_)) should now be reconsidered in the second round. Both types of competitions are Type A, and the two waves around the interface coexist. Finally, the pattern shown in (**a**) becomes stable against iterations described in Discussion, and represents the asymptotic pattern.

To test our results, we take a five-submedium system as an example. The result of pattern formation, dispersion relation curves and driving-response curves of each submedium are plotted in Fig. 8. We observe the coexistence of natural oscillations (in *M*_1_ and *M*_3_) and wave trains (in the other three submedia). The interface between *M*_4_ and *M*_5_ is transparent, whereas others are visible. By considering the dispersion relation curves (Fig. 8(b)) and driving-response curves (Fig. 8(c)), the types of competitions around all of the interfaces can be well identified. For Step 1a), the competition around interface (*M*_1_, *M*_2_) is of Type C (*α*_1_ dominates its own submedia and invades *M*_2_); those around interfaces (*M*_2_, *M*_3_) and (*M*_3_, *M*_4_) are of Type A (coexistence of two natural oscillations), whereas that around interface (*M*_4_, *M*_5_) is of Type B (ISW dominates both *M*_4_ and *M*_5_). Now, in some submedia (i.e., *M*_2_ and *M*_4_), there are two winners from the competitions at the interfaces; the two winners further compete in the same submedia *M*_2_ and *M*_4_ according the rules of Eq. (1) in step 1b), and only one wave is left in each submedium (*α*_1_ in *M*_2_ and ISW in *M*_4_). The story does not end at this stage. One must process to the second round to investigate the interface competitions for Step 2 (interfaces (*M*_2_, *M*_3_) and (*M*_3_, *M*_4_)). Both competitions at the interfaces of the reiterated (*M*_2_, *M*_3_) and (*M*_3_, *M*_4_) are of Type A, and only one winner is left in each submedium in each competition. The pattern now becomes stable for the further iterations, which is the pattern of Fig. 8(a), the final result of Step 3.

## Method

### Properties of Homogeneous CGLE

A homogeneous CGLE medium can support plane wave solutions, and the frequency and wave number of wave trains satisfy the dispersion relation^19^,^27^:


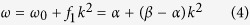


with *f*_1_ = *β* − *α* being the slope of the dispersion relation curve in the *ω* − *k*^2^ plane and *α*, called the natural frequency (*ω*_0_ = *α*) of CGLE, being the actual frequency of the homogeneous no-flux systems. The dispersion relation curve Eq. (4) and the frequency of the wave train (*ω*) determine the characteristic of the propagating waves in the medium^27^:


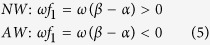


### Numerical Simulation

The CGLE system is integrated using the explicit Euler-method and standard three-point approximation for the Laplacian operator. Moreover, no-flux boundaries are utilized. Throughout the paper, we take *L* = 200 and simulate Eq. (5) using space step Δ*x* = 1.0 and time step Δ*t* = 0.005. In this paper, by considering a multi-medium 1D CGLE system, we study how the simplest heterogeneity can greatly influence the outputs of the system and induce rich complexity.

## Conclusion

In conclusion, we would like to emphasize that many experimentally important oscillatory media are inhomogeneous; thus, wave competitions and pattern formations in heterogeneous systems are of great significance in practical applications. In this paper, we use a multi-medium CGLE system as the simplified example and study the influences of heterogeneity. In the presence of different parameters, the results of such competitions are diverse and interesting. The complexity comes from the diversity of dispersion relations and driving-response curves in different media and the participation of interface-selected wave trains (ISWs) which have not been considered by previous works considering heterogeneity^13^,^14^,^15^,^16^,^17^. The mechanisms underlying the rich behaviors of the wave patterns shown in Figs 1, 2, 3 and 8 have been fully understood and can be well predicted by classifying the A, B, C types of competitions at the interfaces and by following the three typical steps described in the Discussion. It is emphasized that all of the results obtained in this paper are robust for heterogeneous oscillatory media without external pacing. However, many rich and interesting characteristic features and potential applications of heterogeneous oscillatory systems have not yet been explored and understood. Further investigation in this direction may greatly broaden our scope regarding pattern formations, competitions, and the control of oscillatory waves.

## Additional Information

**How to cite this article**: Cui, X. *et al.* Waves spontaneously generated by heterogeneity in oscillatory media. *Sci. Rep.* **6**, 25177; doi: 10.1038/srep25177 (2016).

## Supplementary Material

Supplementary Information
