# Supplementary material for: Waves spontaneously generated by heterogeneity in oscillatory media
Source: Sci Rep. 2016 May 4;6:25177. doi: 10.1038/srep25177 (PMC4855178; doi:10.1038/srep25177)
Supplement: Supplementary Information [file srep25177-s1.pdf]

Supplementary Information
Waves spontaneously generated by heterogeneity in oscillatory media

Xiaohua Cui, Xiaodong Huang and Gang Hu

We plot a diagram showing the regimes where patterns in Figs. 1(a)(b)(c) can be observed. It is clear that all these patterns appear robustly in wide parameter areas. In Fig.S1, the parameter set of M_1 is fixed as $\alpha_1 = -0.15, \beta_1 = 1.0$ and the parameter set of M_2 is varied. The three types of patterns in Fig.1 are denoted by Type A, B and C, which are clearly marked in Fig.S1. Both clear straight lines (the green dotted line and red dashed line) can be deduced from Competitions at Interface. We take Type B (interface-selected waves, ISW, dominating the whole two-medium system) as an example. For the existence of ISW, the dispersion-relation curves of M_1 and M_2 must have slopes of opposite signs. The sign of M_1's dispersion relation curves should be negative as the sign of M_2's are positive, so we have $\beta_2 < \alpha_2$ which is below the green dotted line. Moreover, the two curves must have an intersection, so we have $\alpha_2 > \alpha_1$ which is on the right of the red dashed line. The region of Type B where ISW dominating the two-medium system is in agreement with the distribution of ISW, but it cannot occupy the whole lower left region as there is a limit of each medium's oscillatory frequency.

The wave patterns in three-submedium systems shown in Fig.6 can be fully understood by the similar ideas used in two-medium systems.

In Fig.S2 the dispersion relation curves and driving-response curves of system Figs.3(a)(b) are plotted. As the neighbor submedia M_1 and M_2 have slopes with opposite signs in the $\omega - k^2$ plane, and they intersect, an ISW exists (Type B). This ISW dominates M_1 and M_2, and then competes with the natural oscillation in the third submedium M_3. For parameters of Fig.3(a), this ISW is not allowed by M_3, neither the natural oscillation α_3 is allowed by M_2 (Type A), and finally the coexistence of ISW in M_1 and M_2 and natural oscillation $\omega = \alpha_3$ in M_3 is observed. For parameters of Fig.3(b), the ISW between M_1 and M_2 is allowed by M_3 (Type C), and this ISW defeats the natural oscillation in M_3 due to the rule of Eq.(1), and dominates the whole system with same frequency but different wave numbers in the three submedia. The wave numbers in M_1 and M_2 are equal, but is different in M_3.

In Fig.S3 the dispersion relation curves and driving-response curves of system Figs.3(c) are plotted. The neighbor submedia (M_1 and M_2) and (M_2 and M_3) both have slopes with opposite signs, and intersect (Type B). Two ISW trains are generated at the interfaces between (M_1, M_2) and (M_2, M_3). These two ISWs compete in M_2 by Eq.(1), and the one generated at the interface (M_2, M_3) wins the competition due to the rule of Eq.(1), and dominates the whole system(AWs in M_1 and M_3 while NW in M_2).

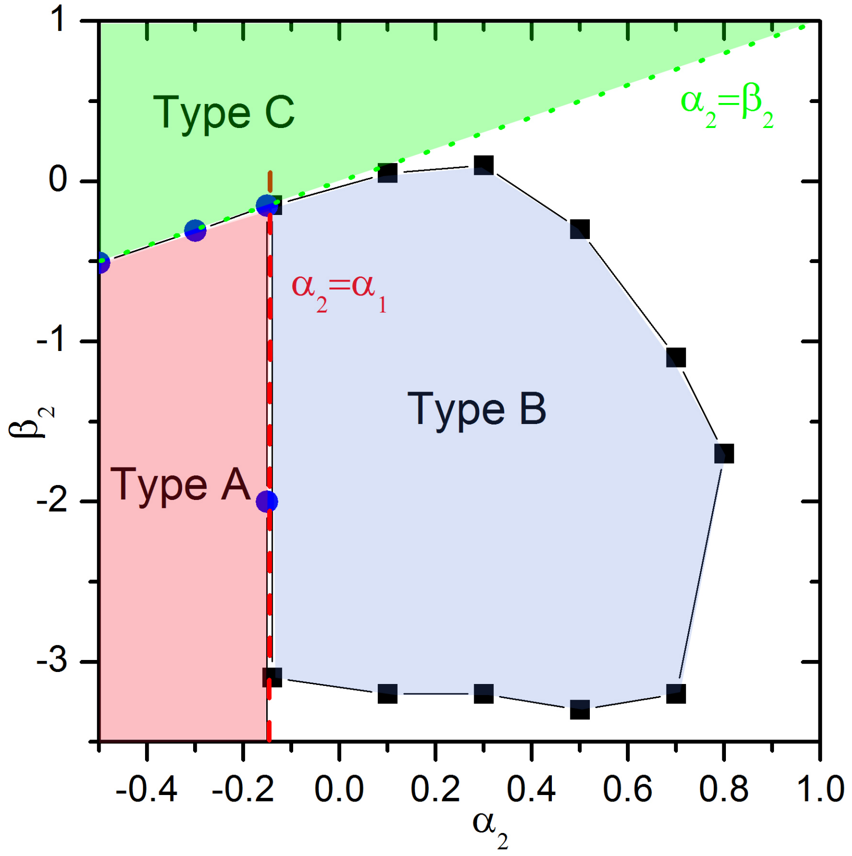

Figure S1. Distributions of different types of wave patterns in α_2, β_2 parameter planes for a set of $\alpha_1 = -0.15, \beta_1 = 1.0$). The red dashed line represents $\alpha_2 = \alpha_1$, and the green dotted line represents $\alpha_2 = \beta_2$. The black boxes represent the boundaries of ISW region (Type B, colored by light blue), the blue dots represent the boundaries of the region where two natural oscillations coexist (Type A, colored by pink), and the other regions (colored by light green and white) are the regions where we observe coexistence of natural oscillation and wave trains. The frequencies of wave trains in white region are different with the coexisting natural oscillations, that is different to the case in region colored by light green (Type C, where the frequencies of nature oscillations and the wave train are the same).

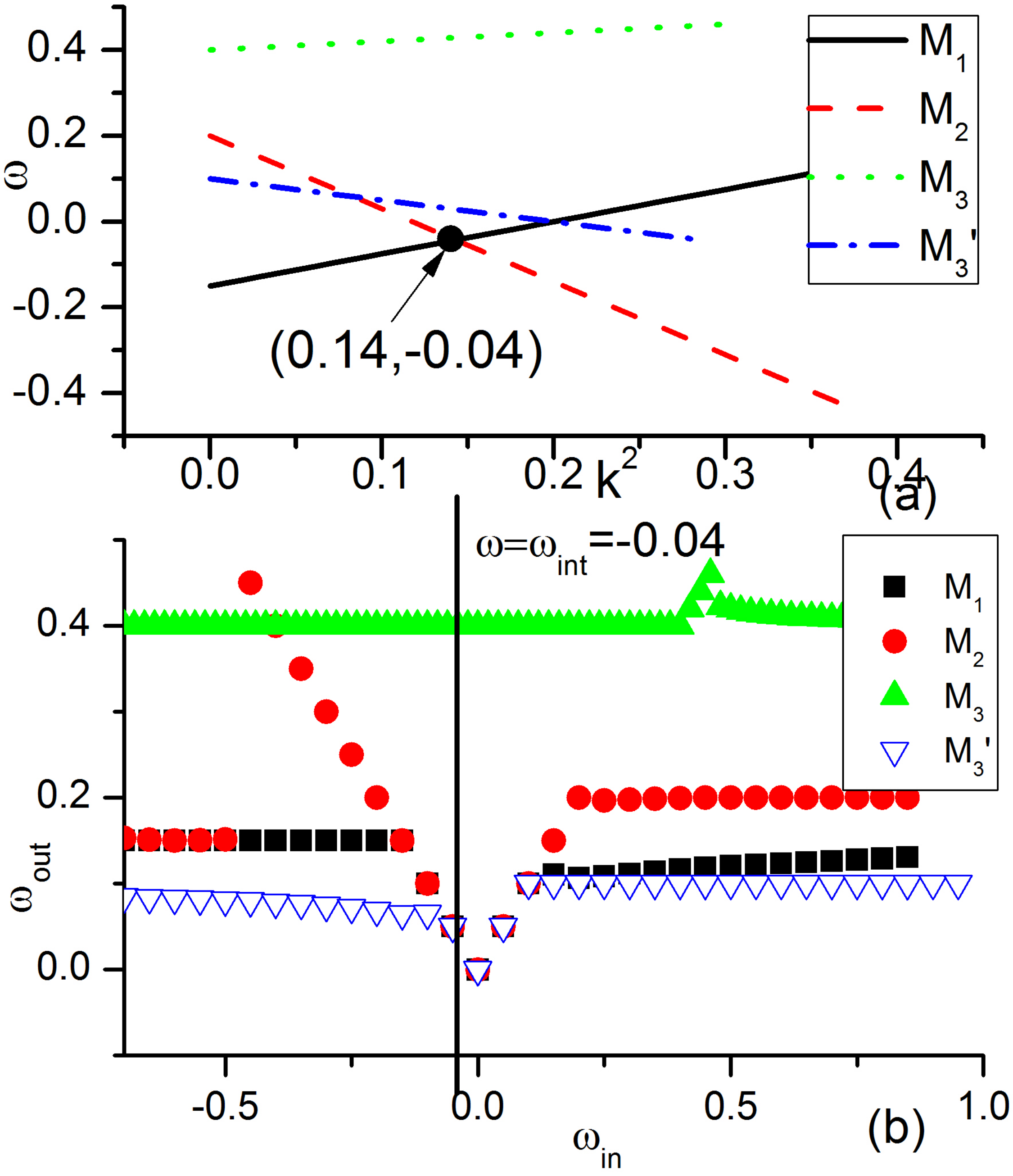

Figure S2. Dispersion relation curves and driving-response curves of systems Figs.3(a)(b). For Fig.3(a) M_1: $\alpha_1 = -0.15, \beta_1 = 0.6$; $\alpha_2 = 0.2, \beta_2 = -1.5$; $\alpha_3 = 0.4, \beta_3 = 0.6$. For Fig.3(b) the parameters of M_1 and M_2 are the same as (a), while M_3 denoted by M_3': $\alpha_3' = 0.1, \beta_3' = -0.4$. (a) Dispersion relation curves of all the submedia. (b) Driving-response curves for these submedia. The neighbor submedia of M_1 and M_2 have slopes with opposite signs, and they intersect at $\omega_{int}^{(1,2)} = -0.04, k_{int}^{2(1,2)} = 0.14$. ISW exists between M_1 and M_2 (Type B). This ISW dominates M_1 and M_2, and then competes with the natural oscillation in the third submedia (M_3, M_3'). For parameters of Fig.6(a), this ISW is not allowed by M_3, neither the natural oscillation α_3 is allowed by M_2 (Type A). Finally one observes coexistence of ISW (in M_1 and M_2) and natural oscillation ($\omega_3 = \alpha_3$) in M_3. For parameters of Fig.6(b), the ISW is allowed by M_3' (Type C), and this ISW defeats the natural oscillation in M_3' according to the rule of Eq.(1), dominates the whole system with same frequency but different wave number in M_3'.

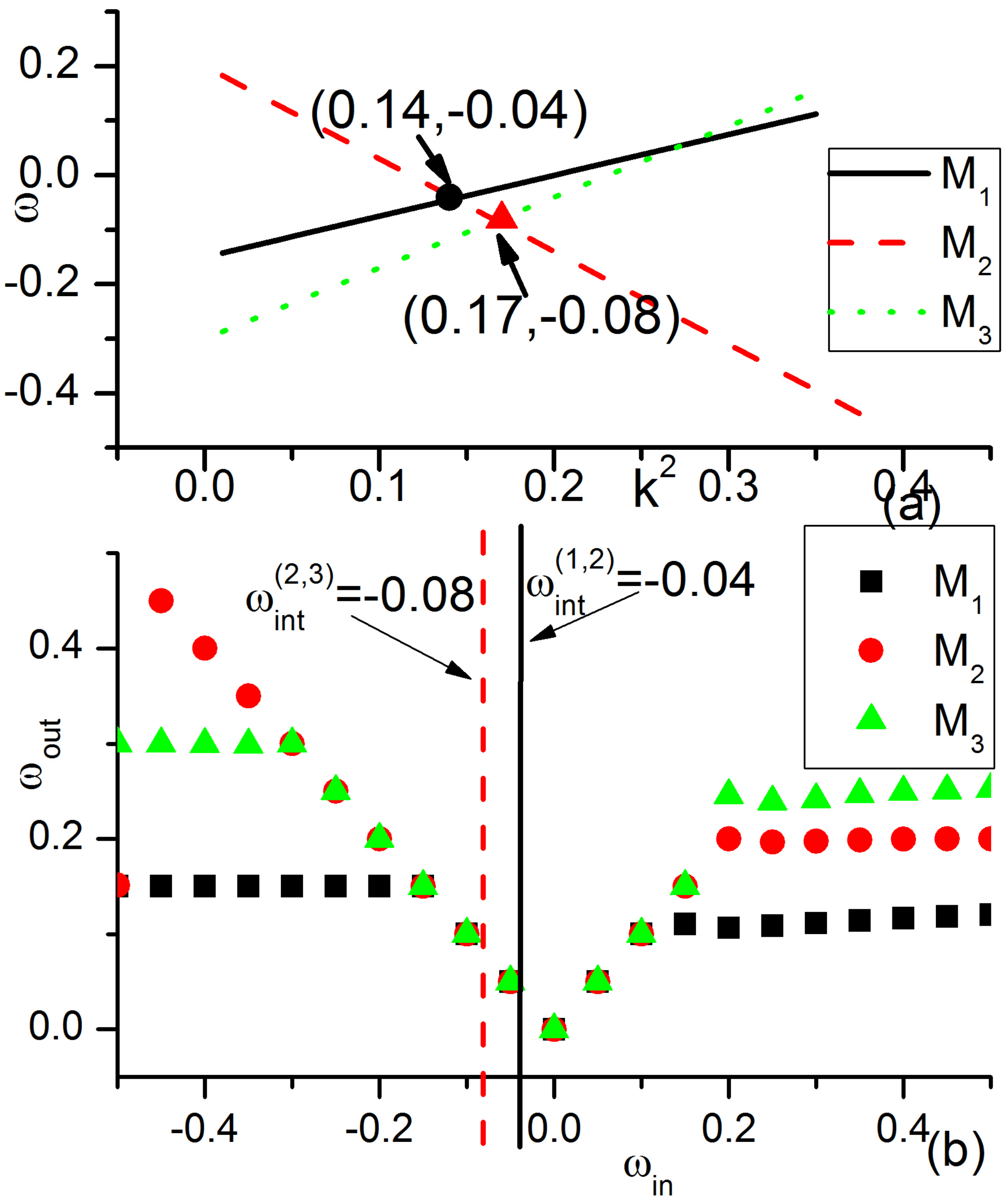

Figure S3. Dispersion relation curves and driving-response curves of system Figs.3(c). M_1: $\alpha_1 = -0.15, \beta_1 = 0.6$; M_2: $\alpha_2 = 0.2, \beta_2 = -1.5$; and M_3: $\alpha_3 = -0.3, \beta_3 = 1.0$. (a) Dispersion relation curves of these submedia. (b) Driving-response curves for the three submedia. The neighbor submedia (M_1, M_2) and (M_2, M_3) both have slopes with opposite signs, and they intersect at $\omega_{int}^{(1,2)}$ for (M_1, M_2) and $\omega_{int}^{(2,3)}$ for (M_2, M_3). Corresponding ISWs exist in (M_1, M_2) and (M_2,M_3) (Type B). These two ISWs compete in M_2 according to the rule of Eq.(1), and the one with frequency $\omega_{int}^{(2,3)}$ wins, dominates M_2 first, crosses the interface of (M_1, M_2), conquers M_1. Finally, ISW with $\omega_{int}^{(2,3)}$ dominates the whole three-medium system(AWs in M_1 and M_3 while NW in M_2). Therefore, one can find transparent interface between (M_2,M_3) while clear interface separation between (M_1,M_2).
